# Can Specialized Pathogens Colonize Distantly Related Hosts? Schistosome Evolution as a Case Study

**DOI:** 10.1371/journal.ppat.0010038

**Published:** 2005-11-25

**Authors:** Sara V Brant, Eric S Loker

Parasites that live in intimate contact with the immune system of their hosts require specialized adaptations to survive in such exposed environments. Once adapted to these demanding environments, it seems such parasites could not easily switch to distantly related hosts [1], and, thus, would be good candidates to diversify congruently with their hosts, i.e., cospeciation. One of the best-known parasite groups is the schistosomes, digenetic (having alternating sexually and asexually reproducing generations in their life cycle) trematodes that live in the vascular system of their vertebrate hosts. Schistosomes achieve notoriety because six of the roughly 100 described species [2,3] cause schistosomiasis, a disease that afflicts 200 million people, mostly in tropical Africa.

Schistosomiasis is usually chronic and debilitating in its course, with most of the pathogenesis caused not directly by the adult worms but by the eggs they produce. Eggs become lodged in the viscera and incite tissue responses, often causing pronounced enlargements in the liver and spleen, and abnormalities in blood flow through these organs. If worms colonize the urinary system, hematuria (blood in the urine) and kidney and bladder damage often result. Schistosomiasis negatively affects growth and productivity, and has largely underappreciated, insidious effects on the people with this disease. Adult worms can be killed by drugs, but the limited availability and high cost of these drugs and the potential for emergence of drug resistance are important concerns. Immunity is slow to develop, though hope for an effective vaccine remains high.

Schistosomes infect birds or mammals, but one species, *Griphobilharzia amoena,* often considered the missing link in schistosome evolution, is known to infect freshwater crocodiles [4]. Schistosomes share the habit of living in a vascular habitat with other trematodes, including the Spirorchiidae of turtles and the Sanguinicolidae of fishes. Worms in these three families have two-host life cycles—a snail host and a vertebrate host—and also share a distinctive tegument, or body covering, consisting of two lipid bilayers instead of the typical single bilayer. The double bilayer is believed to be an adaptation for survival in the immunologically hostile environment of the blood [5]. Schistosomes differ from the other two families of blood flukes, though, by being dioecious (having separate male and female worms) and dimorphic (with the two sexes different in morphology), and by having specialized habitat requirements. The remarkable biology of schistosomes has precipitated considerable discussion regarding their origins and their evolution of dioecy (the change from the typical state in trematodes of being hermaphrodites to a state with separate males and females) [2,4,6–10].

**Figure 1:**
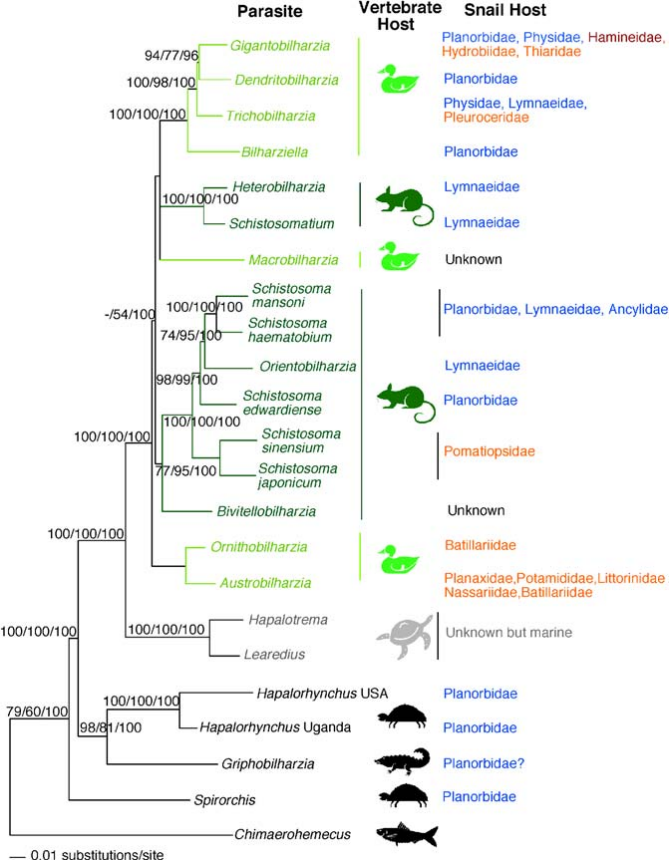
Maximum Likelihood Estimated Tree from the Combined Data Partitions of 6,335 Bases Derived from 18S and 28S Ribosomal DNA and Partial Cytochrome Oxidase I Mitochondrial DNA Genes Nodal support values are indicated on the branch as bootstrap values for maximum parsimony/minimum evolution/Bayesian posterior probabilities [27–28]. To the right of the tree are listed the vertebrate host groups: birds are light green, mammals are dark green, marine turtles, freshwater turtles, freshwater crocodiles, (GenBank accession numbers 28S: AY899914; 18S: AY899915; cytochrome oxidase I: AY899916), and fish are black, and the families of snail hosts used by the various schistosome species are blue indicating families belonging to the Pulmonata, orange to the Caenogastropoda, and red to the Opisthobranchia. At our present state of knowledge, schistosomes that infect birds are not united in a single lineage, nor are those that infect mammals, which is suggestive of at least two separate colonizations of at least one of these lineages. With respect to snail hosts, in at least two instances, even congeneric schistosomes depend on markedly divergent gastropod lineages (pulmonates versus opisthobranchs or caenogastropods), suggesting that, historically, host switching has also been extensive within molluscan hosts [7,13,14,20].

The discovery of *G*. *amoena* [4], the only species of schistosome known in an ectotherm, gave rise to a hypothesis that schistosomes arose in early ectothermic archosaurs, for example, ancestors of modern crocodiles, and radiated into endothermic archosaurs (birds). This view was supported by a phylogenetic analysis of adult morphology, which placed *G*. *amoena* as basal, or ancestral, among schistosomes [11], and challenged the role of endothermy as the pivotal factor driving schistosome diversification [10–12]. Molecular phylogenetic studies to date [7,13,14] have been hampered by an incomplete sampling of the 13 widely recognized schistosome genera, including the provocative and putatively basal *G*. *amoena*. The molecular phylogeny in Figure 1 includes representatives of all the commonly recognized genera of schistosomes, and spirorchiids from both freshwater and marine turtles [7]. Included in this molecular phylogeny is *G*. *amoena,* specimens of which were recovered from the Australian freshwater crocodile, *Crocodylus johnstoni,* obtained near Darwin, Australia.

Our analysis shows that *G*. *amoena* is, in fact, not a basal schistosome, but is more closely related to spirorchiids from freshwater turtles. This expands the host range of spirorchiids to include reptiles other than turtles, and suggests that schistosomes parasitize only endotherms. Our analysis confirms that the sister group to the schistosomes are the spirorchiids from marine turtles [7], and that the basal schistosomes are parasites of marine birds and snails (Figure 1). This pattern supports the idea that a long-range host switch from turtles to avian hosts occurred in marine habitats, and that schistosomes subsequently colonized birds, mammals, and freshwater snails. This argues against a hypothesis of a long-term association between schistosomes and archosaurs (crocodilians), and suggests that exploitation of endotherms has been the key factor leading to the emergence of schistosomes and their dioecious condition [2,8]. We speculate that the transferal of a spirorchiid protoschistosome to an endothermic host was favored by the partial endothermy [15,16] of their ancestral marine turtle hosts.

Endotherms have metabolic rates that are roughly one order of magnitude higher than those of ectotherms of comparable size, and they consequently ingest more food [17]. Nutrients are then conveyed to the liver via the hepatic portal system. Most species of schistosomes live in the hepatic portal system. We argue that schistosomes colonized this specific habitat in endotherms to take advantage of its high nutrient content. In contrast, spirorchiids prefer the heart and arterial system [10], but can be found in widely scattered locations in the vascular system [18,19]. One of the unique demands imposed by the hepatic portal system is the need for the adult worms to move against the flow of blood to reach the small blood vessels surrounding the intestinal wall where oviposition would best lead to expulsion of the eggs in the feces. This requires a difficult combination of strength, to allow movement against the flow of blood, and delicacy, to allow extension of the body into the smallest vessels for oviposition.

One solution is to forego the normal digenean condition of hermaphroditism to relegate each of these functions to separate sexes [2]. The muscular schistosome males are specialized to move females to preferred oviposition sites, whereas the delicate, thread-like females [10] are adapted to insinuate into tiny vessels for oviposition. Precision oviposition is crucial for schistosomes because eggs swept into the liver are not only a dead end from the parasite's point of view, but they also impose significant pathology on their hosts [10].

Schistosomes remain paradoxical because they are specialized in morphology and habitat, and given the intimacy of contact with their host, it seems these worms would become restricted to particular hosts and consequently exhibit cophylogenetic patterns with those hosts. Our results (Figure 1), however, suggest that, historically, schistosomes have undertaken numerous long-range host switches among both vertebrates and snail hosts [7,13,14,20]. Observations that avian schistosomes can persist for a surprisingly long period in mammalian hosts, and likewise for some mammalian schistosomes in avian hosts, provide further evidence for this propensity [21–23].

We propose that schistosomes retain a pleisiomorphic (ancestral trait), nonspecific immune evasion strategy, such that long-distance host switches occasionally occur. The double tegumental membrane and the ability of the tegument to acquire macromolecules from many different host species [5] may contribute to this switching ability. With the possible exception of some species in derived taxa, such as duck schistosomes of the genus *Trichobilharzia* (see Figure 1) [24], the phylogeny of schistosomes does not mirror that of its hosts. Free-swimming larval schistosomes, the miracidia (for snails) and cercariae (for vertebrates), no doubt incessantly attempt to colonize new potential hosts, creating opportunities for host switches. Our results suggest that there are fundamental immune evasive mechanisms dictating schistosome success in intravascular environments in humans and other vertebrate hosts that we do not yet fully appreciate. They also suggest that morphological and microhabitat specialization need not preclude successful colonization of new hosts, indicating that congruent patterns in host and parasite phylogenies may be rare in these specialized worms [25,26].
